# Knowledge, Attitudes, and Practices Associated with Brucellosis in Livestock Owners in Jordan

**DOI:** 10.4269/ajtmh.15-0294

**Published:** 2015-12-09

**Authors:** Imadidden I. Musallam, Mahmoud N. Abo-Shehada, Javier Guitian

**Affiliations:** Department of Production and Population Health, Veterinary Epidemiology, Economics and Public Health Group, The Royal Veterinary College, University of London, Hertfordshire, United Kingdom; Faculty of Epidemiology and Population Health, London School of Hygiene and Tropical Medicine, London, United Kingdom

## Abstract

We evaluated livestock owners' knowledge, attitudes, and practices regarding brucellosis in Jordan. A questionnaire was administered and biological samples were examined to verify the serological status of animals. Seroprevalence estimates indicated that 18.1% (95% CI: 11–25.3) of cattle herds and 34.3% (95% CI: 28.4–40.4) of small ruminant flocks were seropositive. The results showed that 100% of the interviewed livestock keepers were aware of brucellosis: 87% indicated a high risk of infection if unpasteurized milk is consumed and 75% indicated a high risk if unpasteurized dairy products are consumed. Awareness of the risk of infection through direct contact with fetal membranes or via physical contact with infected livestock is considerably lower, 19% and 13%, respectively. These knowledge gaps manifest in a high frequency of high-risk practices such as assisting in animal parturition (62%), disposing aborted fetuses without protective gloves (71.2%) or masks (65%), and not boiling milk before preparation of dairy products (60%). When brucellosis is suspected, basic hygiene practices are often disregarded and suspect animals are freely traded. Public health education should be enhanced as the disease is likely to remain endemic in the ruminant reservoir as long as a suitable compensation program is not established and trust on available vaccines is regained.

## Introduction

Brucellosis is a highly contagious zoonosis affecting humans and a wide range of terrestrial animals.^1^ The disease is caused by members of the genus *Brucella* among which *Brucella melitensis* that mainly infects small ruminants is considered the most virulent species.^2^ Despite a high burden of infection in many areas of the world brucellosis is rarely prioritized by health systems and is considered a neglected zoonosis by the World Health Organization (WHO).^3^

Humans can acquire the infection through consumption of unpasteurized milk or dairy products from infected animals and through direct contact with contaminated tissues or secretions from infected animals, in particular aborted fetuses, fetal membranes, and vaginal discharges.^4^,^5^ As a result, individuals who have occupational contact with livestock in endemic areas are at high risk (e.g., livestock owners, abattoir workers, shepherds, and veterinarians).^4^,^5^ The symptoms of the disease are nonspecific but the majority of patients, in the acute form, complain of fever (over 38.5°C), sweats, malaise, anorexia, headache, arthralgia, and backache. Persistent and recurrent fever are the most common clinical symptoms in subacute cases. Complicated cases may go on to develop arthritis, spondylitis, sacroiliitis, osteomyelitis, meningoencephalitis, and endocarditis.^6^

Brucellosis is endemic in parts of Africa, Central and South America, and Asia, with Middle East countries considered to have the highest incidence of human infection worldwide.^7^,^8^ A recent study in Jordan demonstrated high levels of brucellosis in ruminants, with 18.1% (95% confidence interval [CI]: 11–25.3) of cattle herds and 34.3% (95% CI: 28.4–40.4) of the small ruminant flocks estimated to have seropositive animals (in the absence of vaccination).^9^ The main livestock species in Jordan are sheep, goats, cattle, and camels, with sheep and goats accounting for more than 97% of the total ruminant population, cattle comprising just 2% and camels < 1%.^10^ Sheep and goats are kept in flocks of widely variable size, including small household flocks that are often mixed with other species. Small ruminant production is associated with high levels of human contact, resulting in a high risk of zoonotic disease transmission.^11^ Cattle are mainly reared in intensive or semi-intensive dairy farms, with only small numbers kept as household animals. There is limited information on the incidence of human brucellosis in Jordan but recent studies suggest it may be increasing and that the main risk factors for infection are consumption of milk and dairy products and direct contact with animals.^12^,^13^

Control of brucellosis in ruminants is the key to preventing the disease in humans and can best be achieved through a combination of livestock vaccination, removal of infected animals, and improved hygiene practices that minimize the risk of introducing infection to disease-free flocks/herds.^14^ In highly endemic areas, such as Jordan and the Middle East, basic hygiene practices are of paramount importance in minimizing the risk of disease transmission from livestock to humans. Livestock owners' own knowledge and behavior must be taken into account if sustainable control programs are to be implemented.^15^–^17^ Lack of sufficient knowledge of the disease accompanied by high-risk practices and absence of effective prevention and management strategies result in continuous disease circulation in the population.^18^–^20^ Knowledge, attitudes, and practices (KAP) surveys are a powerful tool in evaluating the vulnerability of livestock owners to livestock diseases, especially in resource-scarce settings.^21^ Moreover, KAP studies have proved valuable to policy makers in helping to develop strategies and health education programs for the prevention of zoonotic diseases such as highly pathogenic avian influenza among rural populations in China.^22^

Previous KAP studies regarding brucellosis among people with high levels of livestock contact in different endemic settings have revealed highly variable results. A study in Kenya has shown poor awareness and knowledge of the transmission routes of brucellosis from animals to humans.^23^ Similarly, poor knowledge and frequent high-risk behaviors regarding brucellosis were observed in a survey of small-scale dairy farms in Tajikistan.^24^ In contrast, a high level of knowledge of the disease was found in a KAP study conducted in a village in the Nile Delta region of Egypt, where despite the high level of awareness and detailed knowledge of disease transmission, high-risk behavior was generalized.^25^

The aim of this study was to assess the KAP of livestock owners regarding brucellosis in Jordan—a middle eastern, low-income country where ruminant brucellosis is present at a high level. It is expected that the results of this study will inform future disease control programs and public health interventions.

## Materials and Methods

### Study design and study population.

Between May and October 2013, a questionnaire survey was conducted among livestock keepers in all the Jordanian governorates (*N* = 12). The study units were small ruminant flocks and cattle herds, randomly selected across the country as part of a cross-sectional study aimed at estimating the flock/herd seroprevalence of ruminant brucellosis in Jordan.^9^ The target population consisted of all small ruminant flocks and cattle herds in the country. The desired number of cattle herds and small ruminant flocks to be sampled to generate herd-/flock-level prevalence estimates with a predefined precision was calculated as:

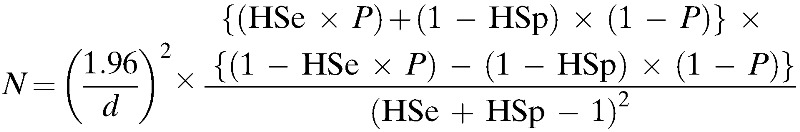
where *N* is the sample size (number of herds/flocks to be sampled), 1.96 is the z-value corresponding to a 95% CI of the standard normal distribution, *d* is the expected absolute error (6%), *P* is the expected seroprevalence at cattle herd or small ruminant flock level (15% and 35%, respectively, based on the most recent estimates available), and HSe and HSp are the herd level sensitivity and specificity of the serological tests used.

The total number of small ruminant flocks (333) and cattle herds (204) was distributed across all the governorates, proportional to their weight within the total population. The serological status of selected herds/flocks with respect to *Brucella* spp. was ascertained by testing a predefined number of individual milk samples (cattle herds) or serum samples (small ruminant flocks) for the presence of antibodies against *Brucella* spp. Full details of the sampling strategy and laboratory procedures are presented elsewhere.^9^ Ethical approval for this study was granted by the Ethics and Welfare Committee of the Royal Veterinary College, London. Informed consent was sought verbally from individual farmers.

### Survey methodology.

A standardized, structured questionnaire (English version available on request from the corresponding author) including mainly close-end questions was used to gather information on livestock owner's KAP concerning brucellosis in animals, potential routes of transmission to humans, and practices regarding dealing with suspected or aborted animals and processing and consumption of milk and dairy products. The questionnaire included two parts: the first part was administered among those individuals responsible for rearing livestock and included questions relating to their knowledge of brucellosis in animal species, clinical signs of brucellosis in ruminants, potential transmission routes from animals to humans, and management of the disease in animals. The second part of the questionnaire was administered among people responsible for the processing of milk and dairy products in the farm/household and aimed to collect information about processing, consumption, and selling of milk and dairy products. The questionnaire from a previous KAP survey on brucellosis in Egypt^25^ was used as a starting point; after consultation among the authors and field veterinarians from the veterinary services of the Jordanian Ministry of Agriculture, the questionnaire was revised and adapted for use in this study and in the Jordanian setting. The resulting questionnaire was piloted by the primary author in 10 farms to confirm that questions and categories were appropriate. As a result of the piloting, modifications were introduced, and the resulting version was used in the survey. It was decided not to include questions about clinical signs of brucellosis in humans because of their nonspecific nature and the diversity of clinical presentations that differ according to the stage of the infection, that is, acute, subacute, and chronic, making the assessment of human disease from questionnaires too complex because of similarities with other acute febrile conditions. Moreover, ruminant brucellosis is endemic in the country, and there is a possibility that most of the livestock owners have developed a mild form of the disease due to the frequent exposure to *Brucella* spp., which may hinder the typical clinical picture of the disease.

Most questions were closed ended with participants asked to choose from a preexisting set of answers: “High risk/Moderate risk/No risk” for questions related to transmission routes from animals to humans; “Most farmers/Some farmers/No one” for questions related to disease management practices; and “Regularly/Sometimes/Never” for questions related to milk and dairy product processing and consumption. Herds/flocks within each governorate were selected by simple random sampling from lists provided by the Ministry of Agriculture. The local veterinarian contacted the owner of each selected herd/flock to explain the purpose of the study. If the owner refused to participate, the next herd/flock owner in the list was contacted until the target sample size for the governorate was reached.

On arrival at the farm/herd/household, the interviewer (a local veterinarian either accompanied by or previously trained by the senior author) explained to the head of the farm/herd/household (or most senior person present at that time) the objectives of the survey, that participation was entirely voluntary, that the identity of the farm/herd/household would not be disclosed, and that biological samples (blood from small ruminants and milk from cattle) will be collected and tested. Following consent to conduct the interview, the person in the farm/herd/household who was mostly responsible for rearing the animals was identified and administered the first part of the interview. When this person was not present, the questionnaire was administered to someone else who regularly looked after the animals. If none of the farm/herd/household members who regularly looked after the animals were available at the time of the visit, the visit was rescheduled. If dairy products were processed in the farm/herd/household, the person responsible for this was interviewed to complete the second part of the questionnaire. If this person was not present, someone else who regularly participated in the processing of dairy products was interviewed, otherwise the visit was rescheduled. In all farms/herds/households that agreed to take part in the study, the questionnaire was administer to someone in charge of rearing the animals (part 1 of the questionnaire) and to someone involved in the production of dairy products (part 2 of the questionnaire). The interviewer recorded the responses of the participants by writing them in Arabic language in a paper copy of the questionnaire precoded with the village and farm identification.

### Data analysis.

The primary author checked the responses registered in the paper version of the questionnaire with the local veterinarian to clarify any possible errors. Then the collected data were stored in Microsoft Access, 2010 (Microsoft, Redmond, WA), by the primary author and was double checked against paper copies for possible entry errors. Descriptive statistics were obtained using Microsoft Excel, 2010 (Microsoft, Redmond, WA). The associations between the serological status of the flock/herd and livestock owner practices when an animal is suspected to have brucellosis, when an animal gives birth, and when an animal aborts were assessed, by means of the χ^2^ test of univariate association. Risk ratios and their 95% CIs were also obtained. A herd or flock was classified as exposed or not exposed to a certain practice based on the answers provided to questions on the “likely course of action of livestock keepers in the village” as opposed to the likely action by the interviewed livestock keeper himself. Associations where deemed significant when *P* < 0.05. The analysis was done using R (3.0.2) (R Development Core Team, 2013).^26^ To visualize whether livestock owners' practices vary geographically, descriptive statistics were also obtained by governorate (*N* = 12) and displayed on a map of Jordan. Only practices carried out by most livestock owners were displayed. Maps were created using ArcGIS 10.2.2 (ESRI, Redlands, CA).

## Results

A total of 537 farms were visited during the study period: 204 cattle herds and 333 small ruminant flocks. On 10 occasions owners refused to participate and were replaced by the following in the list to achieve the desired sample size; number of herds/flocks that was sampled from each governorate is presented in Table 1.

Of the participants in our study, 100% said that they had heard about brucellosis: 49.7% of them from media, 38.6% from local veterinarians, and 11.7% from other farmers.

Around 90% of the participants were sure that sheep can be infected with brucellosis, 62% were sure that goats can be infected with brucellosis, and about 44% were sure that cattle can be infected with brucellosis. On the other hand, more than 55% of the participants were sure that horses, donkeys, poultry, and dogs cannot be infected with brucellosis (Table 2).

When asked about the clinical signs that will be observed in animals that have brucellosis, 76.4% of participants indicated that abortion is the most prominent clinical sign. A considerable proportion of participants also identified difficulties to become pregnant (61.3%), weight loss (59.5%), and drop in milk production (49%). Several other clinical signs were mentioned by a smaller proportion of participants (Table 3).

**Figure 1.**
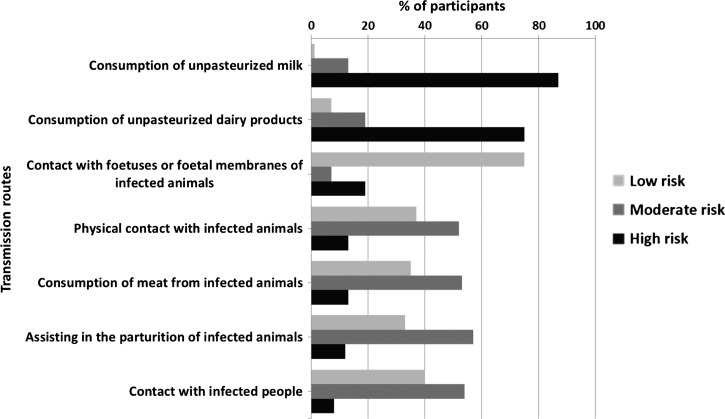
Participants' opinions regarding routes of brucellosis in humans (% of participants considering specific practices to be of low, moderate, or high risk).

Out of 537 participants, 495 (92.2%) declared that they were sure brucellosis can be transmitted from animals to humans, 22 (4.1%) were not sure whether brucellosis can be transmitted from animals to humans, whereas the remaining 3.7% were sure that brucellosis cannot be transmitted from animals to humans. When asked about the level of risk associated with different transmission routes, 87% of the participants indicated that consumption of unpasteurized milk is associated with a high risk of infection. When asked about the consumption of other unpasteurized dairy products, 75% of participants considered it to be a high-risk practice. Conversely, direct transmission routes were perceived to be less dangerous, with less than 19% of the participants considering contact with fetal membranes to be associated with a high risk of infection. Only 10% of respondents considered contact with infected people to be a high risk of infection though more than 50% believed it to be a moderate risk. Participants' views with regard to the risk of human infection associated with different infection routes are presented in Figure 1

.

Participants' opinions regarding the actions that most livestock owners take when they have an infected or suspected animal with brucellosis are presented in Table 4. When asked about how likely it is that farmers sell confirmed or suspected brucellosis cases directly to a neighbor or in the market, 40% of participants agreed that most owners would do this and around 50% agreed that some (but not most) livestock owners would do this, respectively. There is therefore general agreement that infected animals will tend to be sold locally. When asked about the likelihood that these animals are slaughtered at home or sold to a butcher there was considerable disagreement, with similar proportions considering that most farmers would do it and that no one would do it.

Most of the participants also felt that when they have an animal suspected of having brucellosis, most livestock keepers would take measures such as treating the animal, calling the local veterinarian, or separating the animal from others. On the other hand, vaccination does not appear to be an option that most farmers would consider. When asked about practices during parturition of cows, sheep, or goats, more than 60% of the participants stated that most people assist the animal by pulling the calf, lamb, or kid or removing fetal membranes (Table 4). Regarding practices after an abortion: 55% of all participants agreed that most owners will give medications to the animal that has aborted, 50% agreed that most owners will sell the animal in the market, and 31% agreed that the owner will sell the animal to the butcher. Around 85% of the participants believe that no one will vaccinate the aborted animal.

Regarding practices of livestock owners when disposing of aborted fetuses and placental membranes (Table 4): 55%, 52%, and 51% of all participants indicated that feeding an aborted fetus to a dog, disposing of an aborted fetus in a water canal, and disposing of an aborted fetus in the streets, respectively, are practices that most people in the village would conduct. Less than 10% of participants indicated that most owners will burn or bury an aborted fetus. When asked whether livestock owners will wear protective gloves or masks when assisting with the parturition or abortion of animals or while handling placentas and aborted fetuses, less than 6% of all participants believe that most owners will do that (Table 4).

More than 75% of the participants interviewed reported that milk from their own animals was regularly consumed in their household and the same proportion regularly sold raw milk to others. The majority of participants (74%) boil milk before it was consumed. On the other hand less than 40% reported that they boil milk before being processed into yoghurt, cheese, cream, or butter (Table 5).

In the univariate analysis of the associations between livestock owners' practices after abortion, suspicion of infection, or confirmation of infection and the serological status of the herd/flock, those herds/flocks where owners considered separation of the animal as the most common practice were significantly less likely to be seropositive (*P* = 0.03, by χ^2^ test). Similarly, farms in which burying or burning aborted fetuses was considered to be the more likely way of disposal of abortions were at lower risk of infection (*P* < 0.01, by χ^2^ test). On the other hand, herds/flocks where the owner reported butchering an animal that had aborted as the more likely action and those in which the most likely way of disposal of abortions was reported to be feeding it to a dog or throwing it into water canals were at a marginally significantly higher risk of seropositive status (*P* = 0.09 and 0.07, respectively, by χ^2^ test) (Table 6).

There was no marked geographical variation in livestock owner's practices in the event of an infected animal being identified or suspected and in the event of an abortion (maps not presented).

## Discussion

This is the first KAP study regarding brucellosis conducted in Jordan and, to our knowledge, the only such study conducted at a national level using probabilistic selection of herds/flocks and households to achieve whole country representativeness. Our results show that Jordanian livestock keepers are highly aware of brucellosis with 87% indicating a high risk of infection if raw milk is consumed and 75% if unpasteurized dairy products are consumed. On the other hand, awareness of the risk of infection through direct contact with fetal membranes or via physical contact with infected livestock is considerably lower (19% and 13%, respectively). Practices posing a high risk of direct transmission are very frequent: most farmers report that assisting in animal parturition and disposing aborted fetuses without protective gloves or masks are 62%, 71.2%, and 65%, respectively. Despite higher awareness of food-borne transmission routes, practices posing a high risk of food-borne transmission are also common (most farmers reported that not boiling milk before preparation of dairy products was 60%). When brucellosis is suspected, basic hygiene practices are often disregarded and suspect animals are freely traded.

The results of this KAP study together with those of the parallel cross-sectional studies, which demonstrated that the disease is endemic at high levels in both small ruminant and cattle populations in Jordan, provide a strong basis on which the Jordanian national control program for Brucellosis can be revised and public health interventions can be formulated.^14^ Livestock keepers across Jordan are aware of brucellosis, with almost half of them reporting having heard about the disease from media and around 40% from the local veterinarians, which highlights the important role of media and local veterinarians in raising awareness of zoonotic diseases. However, caution should be taken when interpreting the role of local veterinarians, as this may have been overestimated if participants' responses where influenced by the fact that the local veterinarian was administering the questionnaire. Awareness of the disease is generally accompanied by accurate knowledge of the main animal species potentially affected by *Brucella* spp. and the main clinical signs of infection in animals. This detailed knowledge of the clinical signs in animals is consistent with the endemic nature of the disease in Jordan. With regard to routes of human exposure, more than 90% of the participants were sure that brucellosis can be transmitted from animals to humans and that consumption of unpasteurized milk and dairy products poses a moderate or high risk of infection. This level of awareness of the food-borne transmission route contrasts with the low proportion of participants (19%) that considered direct contact with aborted materials, birth fluids, and fetal membranes to be of high risk. When abortions are observed and brucellosis is suspected, key hygiene practices such as separation of animals suspected of being infected and burying or burning of aborted fetuses/placentas appear to be applied only by “some” farmers. Interestingly, univariate analysis revealed associations between the perceived level of implementation of preventive practices and the status of a herd/flock with regard to *Brucella* infection. These results are in accordance with those of the cross-sectional study, in which we showed that farms that implement hygienic practices such as pen disinfection, separating newly added animals, and isolating aborted animals are at lower risk of being seropositive against *Brucella*.^9^ The neglect of these hygiene practices and the use of practices that contribute to the spread and maintenance of infection are the probable cause of the high seroprevalence estimates of ruminant brucellosis seen in Jordan.

Crucially, around 40% of the participants replied that most livestock owners would sell animals suspected of being infected with *Brucella* to neighbors or in the market. Our study strongly suggests that trade of *Brucella*-infected animals is widespread and therefore likely to be a major contributor to the high levels of brucellosis endemicity among ruminants in Jordan, a country where there are no restrictions of animal movement between districts and governorates. The establishment of an animal identification system could allow the implementation of temporary movement restrictions based on the sanitary status of herds/flocks, as well as tracing the movement of individual animals. This would greatly facilitate the effective implementation of the national brucellosis control program. Calling the local veterinarian was not a priority for most livestock owners when they suspect they have animals with brucellosis. This may be due to fear that their animals, if found infected, could be slaughtered without compensation. Similarly, when brucellosis is suspected vaccination is almost never considered as an option, which may reflect the negative perception most livestock owners have of the *B*. *melitensis* Rev 1 live vaccine (the only vaccine currently in use in Jordan and shown to potentially cause abortion when administered to pregnant animals).^27^,^28^

It seems that treating animals that abort or are suspected to have brucellosis is common despite the questionable economic rationale for this, given the cost of the treatment and the fact that fertility of the aborted animal may remain impaired.^29^

When presented geographically, there were no apparent differences across governorates, indicating that the key findings of this study with regard to practices relevant for brucellosis control were fairly homogeneous across the country.

Direct contact with placental membranes and aborted fetuses is a major route of human infection,^4^,^5^ and it was obvious from the participants' answers that most of them were unaware of this route. This lack of knowledge can explain the frequency of high-risk practices, such as assisting in animal parturition or disposing aborted fetuses, performed without wearing protective gloves or masks.

Regarding the risk of infection with *Brucella* spp. through the consumption of raw milk, the results suggest that it is low since more than 73% of the participants reported that they regularly boil milk before consumption. However, the risk of infection is higher through the consumption of other dairy products, with less than 20% of participants reporting that they boil raw milk before making dairy products such as yoghurt, cream, butter, and cheese. This finding is consistent with a previous study,^12^ which concluded that the consumption of locally produced white feta cheese is a significant risk factor for human brucellosis in northern Jordan. The survivability of *Brucella* spp. in different types of dairy products depends on many factors including the type and age of the product, temperature, changes in pH, moisture content, biological action of other bacteria present, and conditions of storage. For example, *Brucella* spp. has been isolated from yoghurt (pH 4.2–4.3) after 2–5 days depending on the fat content of the product.^30^ Other studies demonstrated the presence of *Brucella* spp. in yogurt after 9–22 days depending on the initial concentration of bacteria in the raw milk.^31^ Furthermore, *Brucella* spp. have been isolated from cream, butter, and white cheese after 4, 8, and 20 weeks, respectively.^32^

The WHO recommended that public health education focusing on occupational exposure and consumption of raw milk and other dairy products should be an important part of any brucellosis control program.^4^,^33^ On the basis of our results this is true in Jordan, where the most critical issues to be addressed appear to be occupational exposure of livestock keepers and their families through direct contact with contaminated tissues and food-borne infection of consumers through consumption of dairy products made from unpasteurized milk. One limitation for this study was that, because of their relatively low number and their minimal contribution to the livestock industry in Jordan compared with other livestock populations, camels were not included.

Long-term control of human brucellosis requires reduction of the prevalence of infection in ruminants. Vaccination of small ruminants with *B*. *melitensis* Rev. 1 vaccine is the control strategy adopted by the Jordanian Veterinary Services of the Ministry of Agriculture. However, as shown in the nationwide cross-sectional study that was conducted in parallel with the interviews for this KAP study,^9^ the current vaccination control program in small ruminant flocks achieves very low coverage (of less than 2%). The program does not include cattle vaccination. A revised control program is needed and, given the high baseline prevalence, it is recommended that it should be based on vaccination of small ruminants against *B*. *melitensis*, which is the only species circulating in the country, and cattle are more likely to act as spillover hosts.^25^ The absence of a suitable compensation program for livestock owners (who may have their seropositive animals slaughtered by the authorities) and distrust of vaccines are the main barriers that hamper the establishment of a sustainable ruminants brucellosis control program in Jordan. It is essential that public health education be included in any future brucellosis control program in Jordan.

## Figures and Tables

**Table 1:** Number (%) of small ruminant flocks and cattle herds that were sampled from each governorate in a knowledge, attitudes, and practices (KAP) study carried out between May and October 2013 in Jordan

Governorates	No. (%) of small ruminant flocks	No. (%) of cattle herds
Ajloun	11 (3.3)	10 (4.9)
Amman	34 (10.2)	20 (9.8)
Aqaba	13 (3.9)	1 (0.5)
Balqa	26 (7.8)	16 (7.8)
Irbid	42 (12.6)	41 (20)
Jerash	16 (4.8)	4 (2)
Karak	26 (7.8)	3 (1.5)
Ma'an	42 (12.6)	1 (0.5)
Madaba	13 (3.9)	5 (2.5)
Mafraq	75 (22.5)	24 (11.8)
Tafiela	12 (3.6)	1 (0.5)
Zarqa	23 (7)	78 (38.2)

**Table 2:** Participants' responses regarding animal species that can have brucellosis

Question asked to the interviewed owners	No. (%) of participants						
	Cow	Sheep	Goat	Horse	Donkey	Poultry	Dog
Animal species that can have brucellosis	369 (68.7)	487 (90.7)	494 (92)	33 (6.1)	170 (31.7)	102 (19)	64 (11.9)
Sure that this animal species can have brucellosis	235 (43.8)	483 (89.9)	332 (61.8)	9 (1.7)	21 (3.9)	4 (0.7)	16 (3)
Sure that this animal species cannot have brucellosis	185 (34.5)	25 (4.7)	30 (5.6)	362 (67.4)	458 (85.3)	351 (65.4)	286 (53.3)

Results from 537 livestock owners who participated in a knowledge, attitudes, and practices (KAP) study carried out between May and October 2013 in Jordan.

**Table 3:** Participants' responses regarding clinical signs of brucellosis in ruminants

Clinical signs	No. (%) of participants*
Abortion	410 (76.4)
Difficulties in pregnancy	329 (61.3)
Weight loss	319 (59.4)
Produce less milk	263 (49)
Inflammation of testes	112 (20.9)
Diarrhea	108 (20.1)
Skin lesions	85 (15.8)
Lameness	81 (15.1)
Respiratory symptoms	81 (15.1)
Sudden death	16 (3)

Results from 537 livestock owners participated in a knowledge, attitudes, and practices (KAP) study carried out between May and October 2013 in Jordan.

* Those reporting the clinical sign is observed in animals with brucellosis.

**Table 4:** Participants' responses regarding livestock owner's practices associated to brucellosis in ruminants

	No. (%) of participants		
	Most farmers	Some farmers	No one
Livestock owners' practices when an animal with brucellosis is detected or suspected			
Selling detected animal to neighbors	215 (40)	252 (46.9)	70 (13)
Selling detected animal in the market	215 (40)	274 (51)	48 (8.9)
Giving medications to the detected animal	209 (38.9)	279 (52)	48 (8.9)
Calling the local veterinarian	204 (38)	183 (34.1)	150 (27.9)
Separating detected animal from others	193 (35.9)	188 (35)	156 (29.1)
Selling the detected animal to the butcher	188 (35)	145 (27)	204 (38)
Slaughtering the detected animal in the house	177 (33)	177 (33)	183 (34.1)
Vaccinating detected animal	43 (8)	64 (11.9)	430 (80.1)
Livestock owners' practices when an animal gives birth			
Assisting with parturition	333 (62)	177 (33)	27 (5)
Wearing protective gloves when helping with parturition	30 (5.6)	70 (13)	437 (81.4)
Wearing protective mask when helping with parturition	27 (5)	70 (13)	440 (82)
Livestock owners' practices when an animal aborts			
Feeding aborted fetus to dogs	295 (55)	172 (32)	70 (13)
Giving medications to aborted animal	293 (54.6)	180 (33.5)	64 (11.9)
Throwing aborted fetus in water canals	279 (52)	204 (38)	54 (10)
Throwing aborted fetus in streets	274 (51)	215 (40)	48 (9)
Selling aborted animal in the market	268 (49.9)	206 (38.4)	63 (11.7)
Slaughtering aborted animal the house	262 (48.8)	174 (32.4)	101 (18.8)
Selling aborted animal to neighbors	237 (44.1)	204 (38)	96 (17.9)
Calling the local veterinarian	203 (37.8)	122 (22.7)	212 (39.5)
Selling aborted animal to the butcher	168 (31.3)	190 (35.4)	179 (33.3)
Separating aborted animal from other animals	90 (16.8)	267 (49.7)	180 (33.5)
Burning aborted fetus	48 (9)	279 (51.9)	210 (39.1)
Burying aborted fetus	47 (8.7)	279 (52)	211 (39.3)
Wearing protective gloves when disposing aborted fetus	31 (5.8)	124 (23)	382 (71.2)
Vaccinating aborted animal	29 (5.4)	56 (10.4)	452 (84.2)
Wearing protective mask when disposing aborted fetus	27 (5)	161 (30)	349 (65)

Results from 537 livestock owners participated in a knowledge, attitudes, and practices (KAP) study carried out between May and October 2013 in Jordan.

**Table 5:** Participants' opinions regarding practices related to consumption and processing of dairy products

Livestock owners' practices	No. (%) of participants		
	Regularly	Sometimes	Never
Consume milk produced from your animals	405 (75.4)	78 (14.5)	54 (10.1)
Sell any raw milk	400 (74.5)	68 (12.7)	69 (12.8)
Boil raw milk before consumption	395 (73.6)	97 (18.1)	45 (8.4)
Purchase raw milk from other farmers	200 (37.2)	235 (43.8)	102 (19)
Boil raw milk before making yoghurt	89 (16.6)	127 (23.6)	321 (59.8)
Boil raw milk before making cheese	77 (14.3)	102 (19)	358 (66.7)
Boil raw milk before making cream	68 (12.7)	129 (24)	340 (63.3)
Boil raw milk before making butter	60 (11.2)	152 (28.3)	325 (60.5)

Results from 537 livestock owners participated in a knowledge, attitudes, and practices (KAP) study carried out between May and October 2013 in Jordan.

**Table 6:** Univariate analysis of the association between participants' opinions regarding most livestock owners' practices and the serological status of the herd/flock

Action	Categories	No. of +ve/total (%)	Risk ratio (95% confidence interval)	*P* value
Participants opinions regarding the most common livestock owner practices after identification of brucellosis infected or suspected animals and the serological status of the flock/herd				
Calling the local veterinarian	Yes	125/494 (25)	1.28 (0.58–3.18)	0.58
	No	9/43 (21)		
Vaccinating detected animals	Yes	77/312 (25)	0.97 (0.72–1.31)	0.86
	No	57/225 (25)		
Giving medicines to the detected animal	Yes	124/493 (25)	1.1 (0.63–1.94)	0.74
	No	10/44 (23)		
Selling the detected animal to neighbors	Yes	81/315 (26)	1.1 (0.79–1.45)	0.63
	No	53/222 (24)		
Selling the detected animal in the market	Yes	82/322 (25)	1.05 (0.78–1.42)	0.74
	No	52/215 (24)		
Selling the detected animal to the butcher	Yes	74/300 (25)	0.97 (0.73–1.30)	0.86
	No	60/237 (25)		
Separating the detected animal from others	Yes	53/257 (21)	0.71 (0.52–0. 96)	0.03
	No	81/280 (29)		
Slaughtering the detected animal in the house	Yes	60/237 (25)	1.03 (0.76–1.38)	0.86
	No	74/300 (25)		
Participants' opinions regarding most common livestock owner practices when an animal gives birth and the serological status of the flock/herd				
Assisting with parturition	No	123/489 (25)	1.1 (0.64–1.9)	0.75
	Yes	11/48 (23)		
Wearing protective gloves when helping with parturition	Yes	22/75 (29)	1.21 (0.82–1.78)	0.34
	No	112/462 (24)		
Wearing protective mask when helping with parturition	Yes	41/164 (25)	0.83 (0.51–1.33)	0.43
	No	93/373 (25)		
Participants' opinions regarding the most common livestock owners practices when an animal has aborted and the serological status of the flock/herd				
Calling the local veterinarian	Yes	115/460 (25)	1.01 (0.66–1.54)	0.61
	No	19/77 (25)		
Vaccinating aborted animals	Yes	83/309 (27)	1.2 (0.89–1.63)	0.24
	No	51/228 (22)		
Giving medicines to the aborted animal	Yes	125/511 (24)	0.71 (0.41–1.22)	0.07
	No	9/26 (35)		
Selling the aborted animal to the neighbors	Yes	35/156 (22)	0.86 (0.62–1.21)	0.39
	No	99/381 (26)		
Selling the aborted animal in the market	Yes	127/494 (26)	1.57 (0.78–3.16)	0.09
	No	7/43 (16)		
Selling the aborted animal to the butcher	Yes	120/490 (24)	0.82 (0.52–1.31)	0.42
	No	14/47 (30)		
Separating the aborted animal from other animals	Yes	55/347 (16)	0.38 (0.28–0. 51)	< 0.01
	No	79/190 (42)		
Butchering the aborted animal in the house	Yes	81/286 (28)	1.34 (0.85–2.81)	0.09
	No	53/251 (21)		
Burning aborted fetus	Yes	46/269 (17)	0.52 (0.38–0.71)	< 0.01
	No	88/268 (33)		
Burying aborted fetus	Yes	15/239 (6)	0.16 (0.10–0.26)	< 0.01
	No	119/298 (40)		
Feeding aborted fetus to dogs	Yes	124/468 (26)	1.83 (1.01–3.31)	0.03
	No	10/69 (1)		
Throwing aborted fetus in streets	No	109/429 (5)	1.1 (0.75–1.60)	0.64
	Yes	25/108 (23)		
Throwing aborted fetus in water canals	Yes	114/428 (27)	1.45 (0.95–2.22)	0.07
	No	20/109 (18)		
Wearing protective gloves when disposing of aborted fetus	Yes	60/237 (25)	1.03 (0.76–1.4)	0.86
	No	74/300 (25)		
Wearing a protective mask when disposing of aborted fetus	Yes	41/164 (25)	1.0 (0.73–1.38)	0.2
	No	93/373 (25)		

Results from 537 livestock owners participated in a knowledge, attitudes, and practices (KAP) study carried out between May and October 2013 in Jordan.
